# ApoE4 dysregulation incites depressive symptoms and mitochondrial impairments in mice

**DOI:** 10.1111/jcmm.18160

**Published:** 2024-03-20

**Authors:** Weifen Li, Tahir Ali, Kaiwu He, Chengyou Zheng, Ningning Li, Zhi‐Jian Yu, Shupeng Li

**Affiliations:** ^1^ Department of Infectious Diseases, Huazhong University of Science and Technology Union Shenzhen Hospital Shenzhen University School of Medicine Shenzhen China; ^2^ State Key Laboratory of Oncogenomics, School of Chemical Biology and Biotechnology Peking University Shenzhen Graduate School Shenzhen China; ^3^ Shenzhen Bay Laboratory Shenzhen China; ^4^ Tomas Lindahl Nobel Laureate Laboratory, Precision Medicine Research Centre The Seventh Affiliated Hospital of Sun Yat‐sen University Shenzhen China; ^5^ Campbell Research Institute, Centre for Addiction and Mental Health Toronto Ontario Canada; ^6^ Department of Psychiatry University of Toronto Toronto Ontario Canada

**Keywords:** ApoE4, depression, LPS, melatonin, mitophagy

## Abstract

Apolipoprotein E4 (ApoE4) is involved in the stress‐response processes and is hypothesized to be a risk factor for depression by means of mitochondrial dysfunction. However, their exact roles and underlying mechanisms are largely unknown. ApoE4 transgenic mice (B6. Cg‐*ApoE* ^tm1Unc^ *Cdh18* ^Tg(^GFAP^−APOE i4)1Hol^/J) were subjected to stress (lipopolysaccharides, LPS) to elucidate the aetiology of ApoE4‐induced depression. LPS treatment significantly aggravated depression‐like behaviours, concurrent with neuroinflammation and impaired mitochondrial changes, and melatonin/Urolithin A (UA) + 5‐aminoimidazole‐4‐carboxamide 1‐β‐D‐ribofuranoside (AICAR) reversed these effects in ApoE4 mice. Concurrently, ApoE4 mice exhibited mitophagy deficits, which could be further exacerbated by LPS stimulation, as demonstrated by reduced Atg5, Beclin‐1 and Parkin levels, while PINK1 levels were increased. However, these changes were reversed by melatonin treatment. Additionally, proteomic profiling suggested mitochondria‐related signalling and network changes in ApoE4 mice, which may underlie the exaggerated response to LPS. Furthermore, HEK 293T cells transfected with ApoE4 showed mitochondria‐associated protein and mitophagy defects, including PGC‐1α, TFAM, p‐AMPKα, PINK1 and LC3B impairments. Additionally, it aggravates mitochondrial impairment (particularly mitophagy), which can be attenuated by triggering autophagy. Collectively, ApoE4 dysregulation enhanced depressive behaviour upon LPS stimulation.

## 1 INTRODUCTION

Major depressive disorder (MDD) is a global clinical illness that affects one's mood, thoughts and physical health.^1^, ^2^ Numerous biological, psychological, genetic, social and family factors contribute to the aetiology of depression.^3^, ^4^ Accumulating evidence suggests that inflammation plays a vital role in depression by affecting organism behaviour through several mechanisms,^5^, ^6^ including enhanced proinflammatory cytokines,^1^ abnormal hypothalamic–pituitary–adrenal (HPA) axis due to inflammasomes,^7^ and reduced brain‐derived neurotrophic factor (BDNF) levels in the central nervous system (CNS) induced by innate immunity stress.^7^ However, the exact mechanism and causal relationship between dysregulated inflammation associated with apolipoprotein E4 (ApoE4) and the pathophysiology of depression are yet to be elucidated.

The ApoE family of proteins is a primary lipid and cholesterol transporter mainly expressed by brain astrocytes. However, microglia and neurons can also produce these proteins under stress conditions.^8^, ^9^ ApoE, especially the *ApoE 4* gene, is a vital genetic risk factor for neurodegenerative disorders. It causes neuroinflammation and dysregulates cytokine production.^9^, ^10^ Furthermore, mice expressing humanized *ApoE4* showed increased proinflammatory cytokine levels after LPS treatment,^11^ evidencing its role in inflammation. However, the underlying molecular mechanisms have not been sufficiently elucidated. Moreover, discrepant reports on population studies have separately evaluated ApoE ε4 allele susceptibility to depression, either with positive associations,^12^ additional risks,^3^ or no prominent influences on depression.^13^ Recent longitudinal studies employing a large population have also shown apparent risk effects of ApoE*ε4 for increased depressive symptomology and incident depression status.^14^ These pieces of evidence, amid mixed reports of no association between ApoE*ε4 and depression, indicated a need to elucidate the underlying mechanisms of ApoE4 in the aetiology of depression.^15^

Energy impairment is also evident under chronic stress conditions, which may be the basis for linking mitochondrial damage to depressive disorders.^16^ ApoE4 has also been associated with dynamic mitochondrial impairment; however, the underlying mechanism is yet to be elucidated in MDDs. Mitochondria generate ATP through an electron transport chain operated by electron‐carrier enzyme complexes (I, II, III, IV and V).^17^ Concurrently, itochondrial dynamic networks regulated by associated proteins, including MFN1, OPA1, DRP1, TFAM, PGC1 and Fis1, maintain mitochondrial integrity and quality through fission, fusion and mitophagy.^18^ Uncertainty in this dynamic network can lead to mitochondrial dysfunction, as observed in neurological disorders.^19^, ^20^ AMPK signalling plays a dual role in mitochondria. It is involved in mitochondrial biogenesis and can trigger the destruction of defective mitochondria.^21^, ^22^ Impairments in downstream oxidative and autophagy signalling are primarily involved in mitochondrial dysfunction.^23^ Thus, whether mitochondrial deficits participate in the pathological processes of ApoE4‐related depression is under investigation^24^ but requires further study.

In this study, we treated humanized ApoE4 transgenic mice with LPS to elucidate the role of ApoE4 in neuroinflammation‐associated depression. Our results showed that ApoE4 overexpression increased susceptibility to depression under stress conditions via mitochondrial impairment. We found that LPS treatment enhanced cytokine production, elevated free radical generation, induced mitochondrial network deficits and impaired mitophagy, accompanied by exaggerated depression‐like behaviours in ApoE4 mice. However, melatonin (an antidepressant and an autophagy inducer) reversed these effects.

## 2 MATERIALS AND METHODS

### 2.1 Animals and cells

ApoE4 mice (B6. Cg‐*ApoE* ^tm1Unc^ *Cdh18* ^Tg(^GFAP^−APOE i4)1Hol^/J, No. 004631) with a C57BL/6 background were obtained from The Jackson Laboratory.^25^ Wild‐type (WT.) C57BL/6J mice were purchased from Guangdong Medical Laboratory Animal Center, China. All experimental animals were 12–14 weeks old. The experimental animals were housed at the Laboratory Animal Research Center, Peking University Shenzhen Graduate School, under a 12 light–dark cycle at 18–22°C. The mice had free access to food and tap water throughout the study period. Experimental procedures were designed so as to minimize the suffering of the mice. All the experiment procedures were carried out according to the protocols approved by the Institutional Animal Care and Use Committee of the Peking University Shenzhen Graduate School (Approval Number: 11110).

Human 293T cell lines were grown in high‐glucose Dulbecco's modified Eagle's medium (DMEM) supplemented with 10% foetal bovine serum (FBS) (Gibco, Waltham, MA, USA). The cells were maintained in a humidified incubator with 95% air and a 5% CO_2_ atmosphere at 37°C. When the cells attained 70% confluence, ApoE4 plasmid transfection and drug treatments were performed; subsequently, the cells were harvested and their fluorescence was observed.

### 2.2 Drug administration and schedule

**FIGURE 1:**
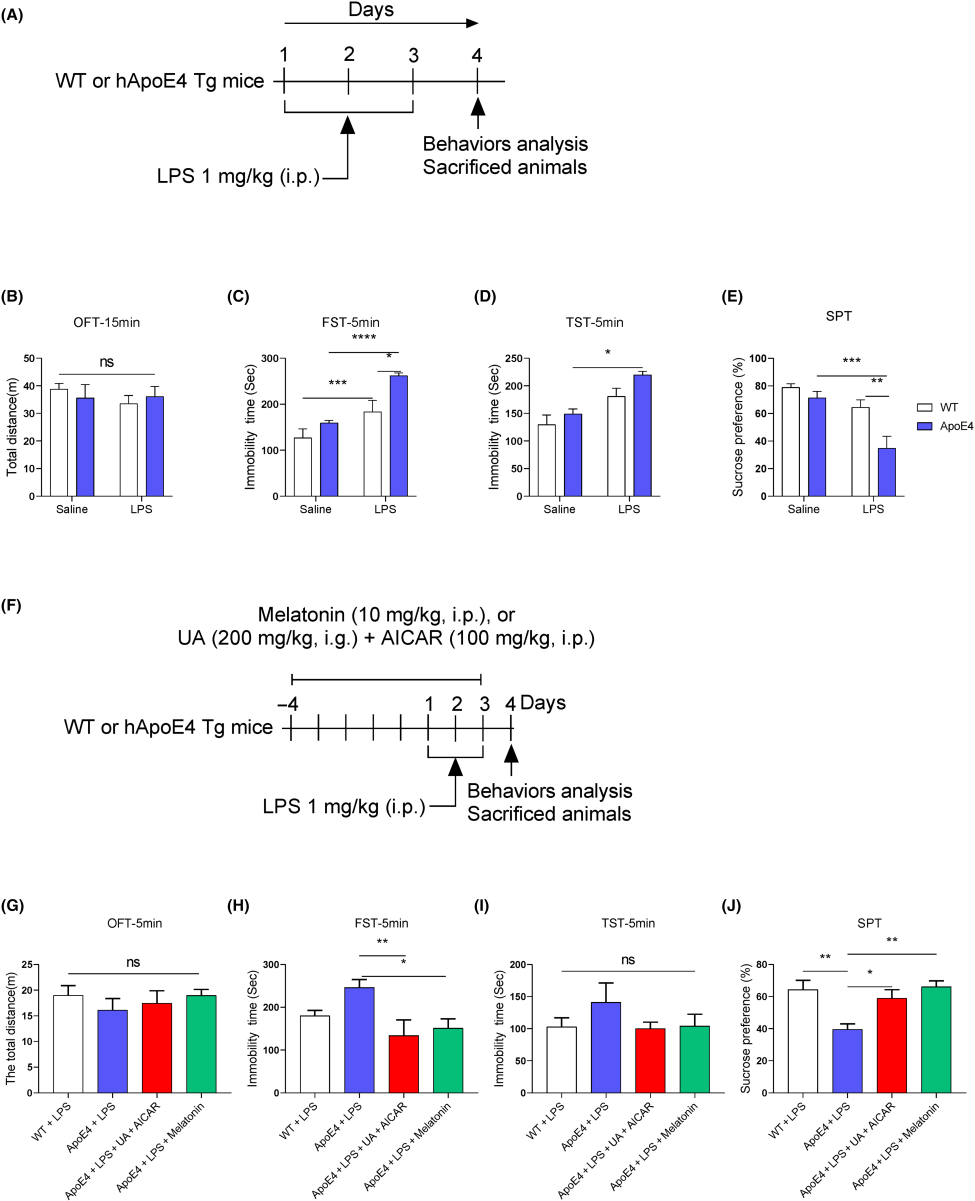
LPS aggravated depressive‐like behaviours in ApoE4 mice. (A/F) Experimental approach; (B/G) OFT; (C/H) FST; (D/I) TST; (E). (F/J) SPT. *n* = 8–11, mean ± SEM, ANOVA, Tukey's test. *p* < 0.05. *p < 0.05, **p < 0.01, ***p < 0.001, ****p < 0.0001. ns = Non‐significant.

The present investigation comprised the following experiments:

First experiment: Group 1: Wt treated with saline (100 μL/10 g). Group 2: Wt treated with LPS (1 mg/kg, i.p.), Group 3: ApoE4 Treated with saline (100 μL/10 g). Group 4: ApoE4 was treated with LPS (1 mg/kg, i.p.) (Figure 1A). LPS were treated for 3 days, 3 doses.

Second experiment: Group 1: Wt treated with LPS. Group 2: ApoE4 treated with LPS. Group3: ApoE4 mice treated with urolithin A (UA) (200 μg/kg, i.g.) + 5‐aminoimidazole‐4‐carboxamide 1‐β‐D‐ribofuranoside (AICAR) (100 mg/kg, i.p.). Group 4: ApoE4 was treated with LPS+ melatonin (10 mg/kg, i.p.) once daily for 2 h before LPS administration (Figure 1F).

Third experiment: Group 1: Wt treated with melatonin (10 mg/kg, i.p.). Group 2: Wt treated with melatonin + Luzindole (5 mg/kg, i.p.). Group 3: ApoE4 treated with melatonin (10 mg/kg, i.p.), Group: ApoE4 treated with melatonin + Luzindole (Figure 9A).

Additionally, ApoE4 and WT mice were administered melatonin (10 mg/kg, i.p.) in separate experiments.

All behavioural analyses (SPT, TST and FST) were performed as reported.^26^, ^27^ 24 h after the final LPS administration. Finally, the animals were sacrificed, and the serum and tissues were collected for further analysis.

### 2.3 Animal behaviour assay

#### 2.3.1 Open field test

To eliminate animal sickness factors and avoid biases due to sickness and blunted behaviours induced by LPS, we performed an open field test (OFT) according to previously developed protocols.^28^ Briefly, the mice were allowed to adapt to the experimental room for 1 h and placed in a 45 × 45 × 30 cm chamber. Fifteen videos were recorded to observe the locomotor activity of the mice. The total distance covered by the mice was measured in metres.

#### 2.3.2 Sucrose preference test

The sucrose preference test was performed using a two‐bottle free‐choice paradigm. The mice were habituated to a 1% sucrose solution for 3 days and then randomly grouped. The mice were deprived of water and food for 24 h and on the 3rd day of drug administration to analyse individual sucrose intake. Each mouse had free access to two sucrose bottles and water bottles the following day. The positions of the water‐ and sucrose‐containing bottles were changed after 12 h. Finally, the volume of water and sucrose solution consumed was recorded and calculated using the following formula:

$$\text{Sucrose Preference}=\frac{\text{Sucrose consumption}}{\text{water and Sucrose consumtion}}\times 100\%$$

#### 2.3.3 Forced swimming test

The forced swimming test (FST) was performed according to our previously developed protocols.^29^, ^30^ First, the mice were trained to swim and a pre‐experiment FST was performed to select healthy and normal mice. During the FST, mice were placed in a Plexiglas cylinder (height: 70 cm, diameter: 30 cm) filled with water above the 30 cm level at 23 ± 1°C. In 6 min of video, the last 5 min were blindly analysed. The mice were considered immobile when they remained motionless in the water and moved to keep their noses above the water's surface. The horizontal movement of the animals throughout the cylinder was defined as swimming, whereas vertical movement against the wall of the cylinder was defined as climbing. EthoVision XT was used to record videos and analyse the results.

#### 2.3.4 Tail suspension test

The tail suspension test (TST) was performed as described previously.^30^, ^31^ Briefly, the mice were individually suspended approximately 40 cm above the floor by their tail with tape in a rectangular compartment (55 [height] × 20 [width] × 11.5 [depth] cm). The immobility time was scored for the first 4 min of the 5‐min video. The EthoVision XT software (Noldus, Netherlands) was used to record and analyse the data.

### 2.4 Proteomic analysis

A proteomic analysis was performed as we have done previously.^32^

### 2.5 NO and H_2_O_2_ measurement and TBARS assay, ELISA, mitochondrial copy number and ATP

Details have been mentioned in the Data S1.

### 2.6 Western blot

Immunoblotting was performed according to a previously described laboratory protocol. The detailed protocol is described in the Data S1.

### 2.7 Statistical analysis

Statistically significant differences among the experimental groups were determined using GraphPad Prism 8 software. An unpaired *t*‐test was used to compare the two groups, while one‐way analysis of variance (ANOVA), followed by Tukey's post‐hoc and two‐way ANOVA, was performed for multi‐group comparisons. Data are presented as mean ± SEM, and *p* < 0.05 was considered as significant.

## 3 RESULTS

### 3.1 LPS aggravated depressive‐like behaviours and neuroinflammation in ApoE4 transgenic mice

ApoE4 is a risk factor for neuropsychiatric disorders, including depression^14^, ^33^, ^34^, ^35^; however, its underlying mechanism has not been addressed. To explore whether humanized ApoE4 induces depression‐like behaviour in mice, we analysed mice behaviour with OFT, FST, TST and SPT. As shown in Figure 1B–E, ApoE4 mice did not show any significant behavioural changes. However, after LPS administration, mice displayed aggravated depression‐like behaviours, as demonstrated by increased immobility and reduced sucrose preference compared to LPS‐treated control mice. These findings indicate that ApoE4 overexpression could elicit more severe depressive‐like behaviours upon stimulation by stressors (like LPS). We validated this hypothesis by treating ApoE4 mice with melatonin (before LPS administration) in the presence of LPS (Figure 1F). Interestingly, melatonin treatment rescued the LPS‐stimulated depression‐like behaviour in ApoE4 transgenic mice (Figure 1G–J). Similar results were also observed with UA and AICAR treatment, activators of mitochondrial biogenesis and mitophagy.^36^, ^37^

**FIGURE 2:**
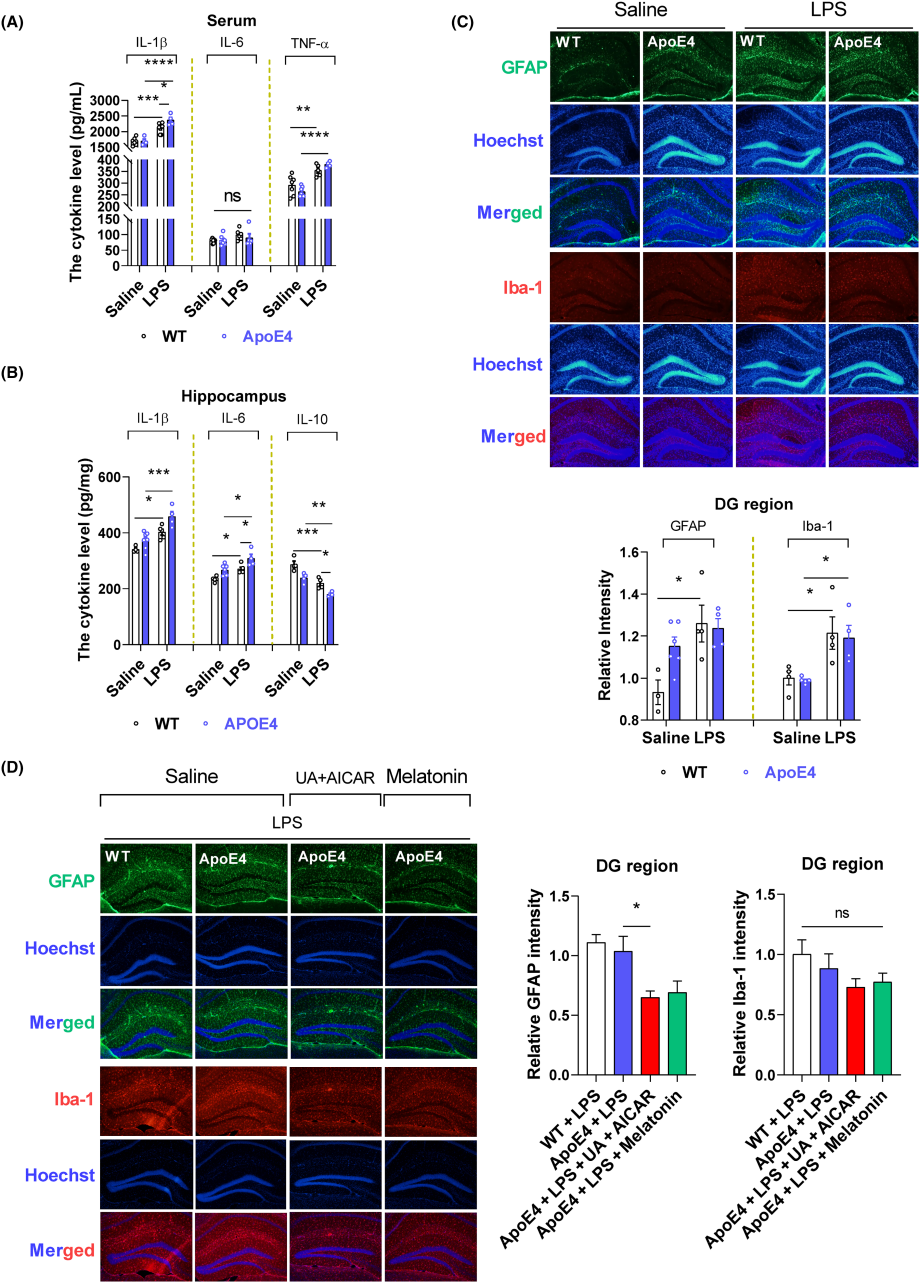
Neuroinflammation in ApoE4 mice. (A) Cytokines, including IL‐1β, IL‐6 and TNF‐α in serum, (B) IL‐1β, IL‐6 and IL‐10, in the hippocampus. In LPS‐treated ApoE4 mice, *n* = 8–10, ELISA. (C) Representative images of immunofluorescence and quantitative bar graphs show expression patterns of GFAP in the LPS‐treated ApoE4 mice. (D) GFAP expression in the hippocampus of the melatonin, UA and AICAR‐treated ApoE4 mice, in the presence of LPS, by immunofluorescence. Bar graphs show relative GFAP in the hippocampus of the mice's brain. *n* = 4–6. Mean ± SEM, ANOVA, Tukey's test. *p* < 0.05. *p < 0.05, **p < 0.01, ***p < 0.001, ****p < 0.0001. ns = Non‐significant.

Neuroinflammation coincides with depression, and LPS is a well‐known inflammatory agent.^38^, ^39^, ^40^ Herein, we determined whether ApoE4 acts as a proinflammatory agent in LPS‐induced depression. Hence, we measured neuroinflammation in the experimental mice (with or without LPS administration). No significant differences were observed in cytokine levels between Wt and ApoE4 mice (Figure 2A,B). However, LPS administration enhanced cytokine (serum IL‐1β, TNF‐α, hippocampal IL‐1β and IL‐6) production in ApoE4 mice than in Wt mice, whereas IL‐10 was significantly reduced in LPS‐treated ApoE4 mice as compared to the WT controls (Figure 2A,B). Proinflammatory cytokines accelerate neuroinflammatory signalling through glial cell activation.^39^, ^41^ We investigated whether LPS‐induced hyperinflammation caused glial cell activation. LPS administration significantly enhanced ionized Ca^2+^‐binding adapter‐1 (Iba‐1), but not glial fibrillary acidic protein (GFAP) expression, in ApoE4 as well as in the Wt mouse hippocampus compared to saline‐treated mice (Figure 2C). However, melatonin and UA + AICAR treatments reduced GFAP expression compared to that in LPS‐treated ApoE4 mice. In contrast, no significant decline in Iba‐1 level was observed after melatonin/UA + AICAR treatment (Figure 2D). GFAP is a major intermediate astrocytic cytoskeletal protein and is considered a marker of astrogliosis, whereas Iba1 is expressed in microglia and is upregulated during the activation of these cells.^26^, ^42^

Mitochondrial abnormalities are accompanied by excessive free radical generation,^43^ which may play a critical role in the pathological process and regulation of depression.^44^, ^45^, ^46^ ROS and NO levels were measured to examine whether the LPS‐induced changes in ApoE4 mice were associated with oxidative and nitrosative stress. LPS treatment significantly enhanced total ROS levels (serum and hippocampus), H_2_O_2_ concentration (serum and hippocampus), hippocampal TBARS levels and NO levels in ApoE4 mice compared to Wt mice, which were attenuated by melatonin administration. Additionally, we examined increased levels of ROS/H_2_O_2_ (serum) and NO in ApoE4 mice compared to those in Wt mice. However, no significant difference in ROS/H_2_O_2_ levels was observed between LP‐treated Wt and ApoE4 mice. In contrast, LPS treatment elevated the ROS/H_2_O_2_/NO levels in ApoE4 mice. Free radical elevation was further validated using TBARS measurements (Figure S1).

### 3.2 LPS‐altered mitochondrial‐associated signalling in ApoE4 mice hippocampus

**FIGURE 3:**
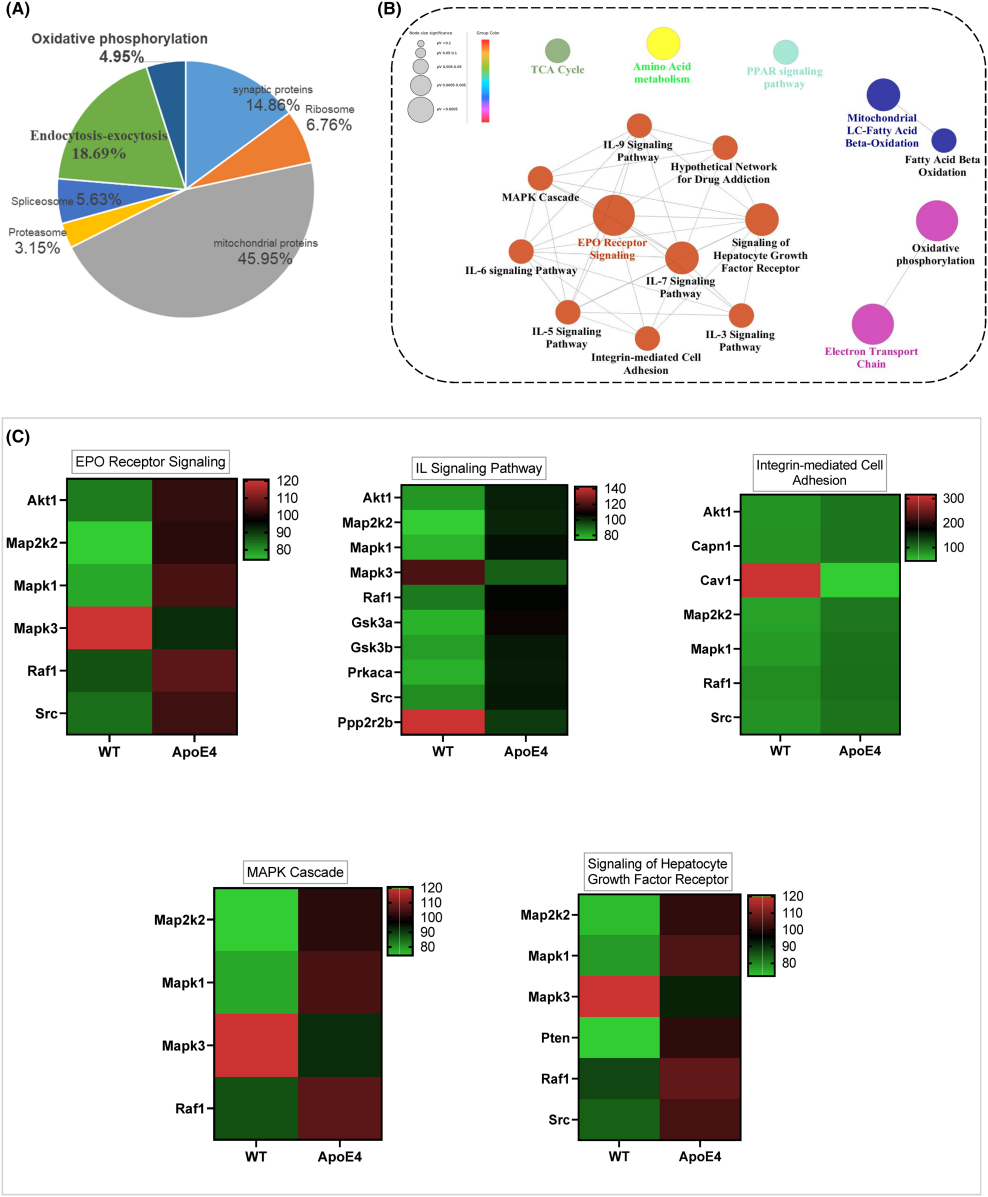
Proteomics analysis of differentially expressed proteins in the hippocampus of ApoE4 mice compared with the WT mice. (A) Functional categories of the differentially expressed proteins in the hippocampus of ApoE4 mice compared with the WT mice. (B) KEGG pathway enrichment analysis of differentially expressed mitochondrial proteins. (C) Heatmap analysis of differentially expressed mitochondrial proteins involved in inflammation‐related terms. Red represents upregulated, and green represents down‐regulated. The brighter the image, the more significant the fold change.

**FIGURE 4:**
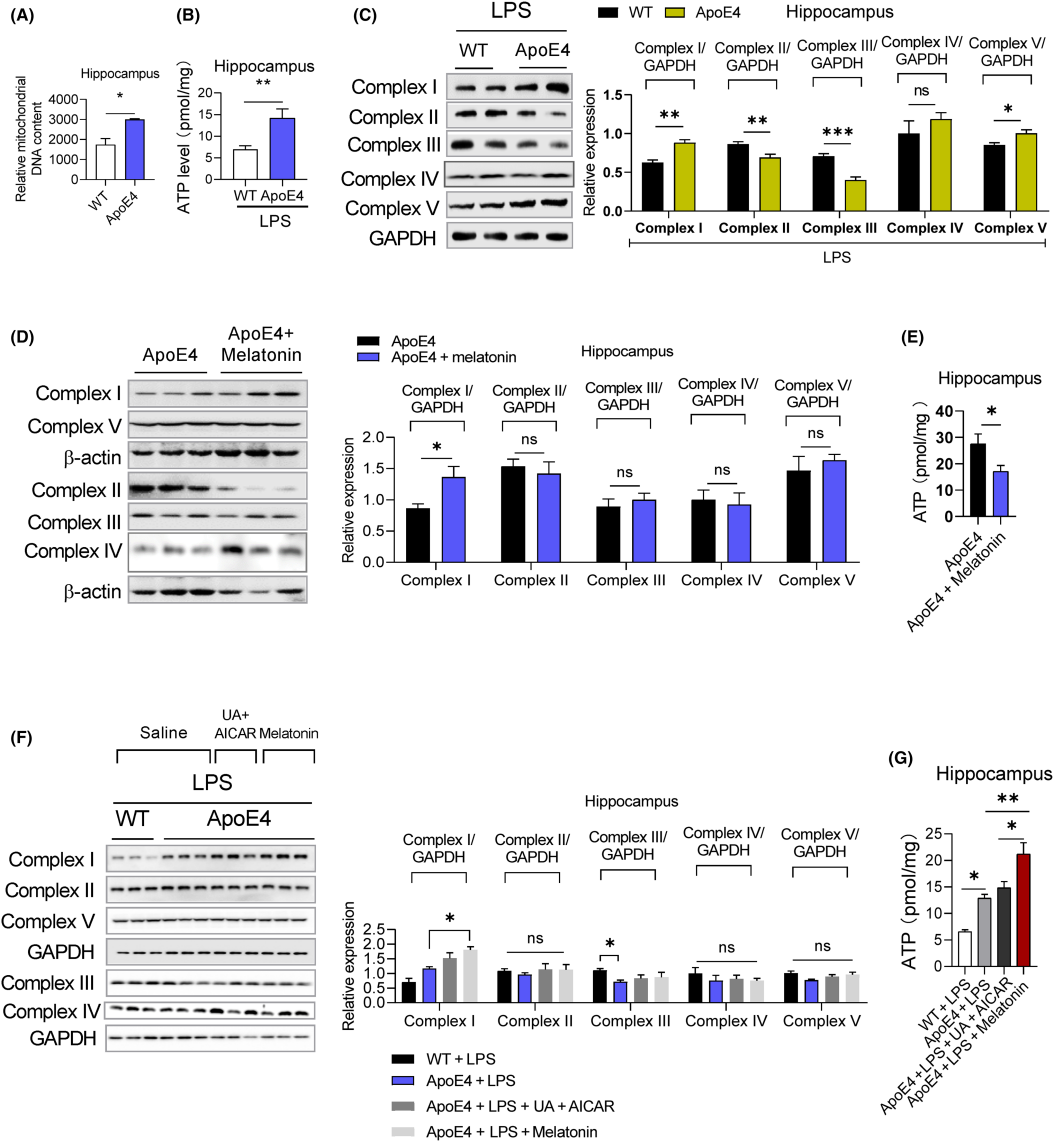
LPS‐altered mitochondrial‐associated proteins in ApoE4 mice. (A) mtDNA content, *n* = 5, unpaired *t*‐test, mean ± SEM. (B/E/G) ATP level, *n* = 5, unpaired *t*‐test, mean ± SEM. (C) Representative immunoblots and bar graphs representing mitochondrial complex proteins expression in the hippocampus of WT/ApoE4 mice, treated with LPS, *n* = 6, unpaired *t*‐test, mean ± SEM. (D) Immunoblot images and column graphs represent the relative expression of mitochondrial complex proteins in melatonin‐treated WT and ApoE4 mice, *n* = 6. (F): Immunoblot images and bar graphs representing the relative expression of mitochondrial complex proteins in melatonin‐, UA + AICAR‐treated ApoE4 mice in the presence of LPS, *n* = 6. ANOVA, mean ± SEM. *p* < 0.05. *p < 0.05, **p < 0.01, ***p < 0.001, ****p < 0.0001. ns = Non‐significant.

Mitochondrial dysfunction is associated with depression,^19^ and previous studies, including ours, have demonstrated that ApoE4 overexpression causes mitochondrial dysfunction.^32^, ^47^ However, the underlying mechanisms and the exact contribution of ApoE4 to MDD are yet to be elucidated. We first performed proteomic profiling to elucidate mitochondria‐associated gene changes in ApoE4 mice (Figure 3).^32^ Mitochondrial DNA content, ATP levels and mitochondria‐associated protein expression were measured in the experimental mice. ApoE4 mice displayed increased mtDNA (Figure 4A) and ATP (Figure 4B) levels, which were reduced by melatonin treatment (Figure 4E). However, LPS treatment increased ATP levels in the hippocampus of ApoE4 mice (Figure 4B), but melatonin and UA + AICAR treatment did not reduce ATP levels (Figure 4G).

To further evaluate ApoE4‐associated mitochondrial dysfunction, we measured the mitochondrial complex protein expression levels. LPS treatment significantly increased complexes I and V and decreased complexes II and III, but did not significantly alter complex IV expression in ApoE4 mice (Figure 4C). melatonin increased complex I expression but did not significantly change the expressions of complex II, III, IV or V, while reducing ATP levels in the hippocampus of ApoE4 mice (Figure 4E). Additionally, melatonin and UA + AICAR combination treatment did not modify mitochondrial complex protein expression in LPS‐treated ApoE4 mice (Figure 4F).

**FIGURE 5:**
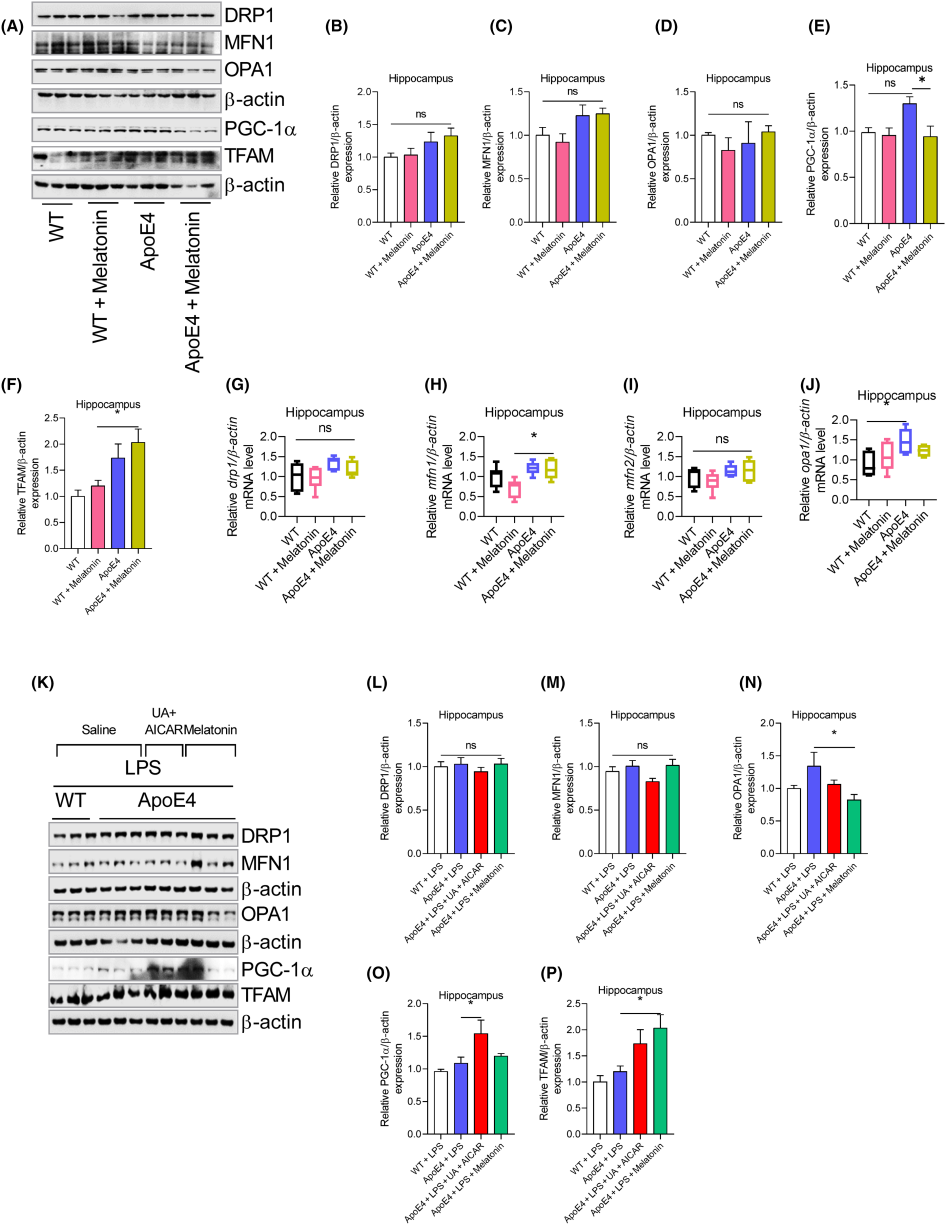
LPS effects on mitochondria‐associated proteins in the hippocampus of ApoE4 mice. (A–F) Immunoblots images and column graphs representing the relative expressions of MFN1, DRP1, OPA1, PGC‐1α and TFAM in the hippocampus of melatonin‐treated WT and ApoE4 mice, *n* = 6. (G–J) Relative mRNA level of drp1, mnf1, mfn2, opa1, in melatonin‐treated WT and ApoE4 mice. (K–P) Representative immunoblot images and bar graphs show DRP1, MFN1, OPA1, PGC‐1α and TFAM expression in melatonin, UA + AICAR‐treated ApoE4 mice in the presence of LPS. *n* = 6. ANOVA, mean ± SEM. *p* < 0.05. *p < 0.05, **p < 0.01, ***p < 0.001, ****p < 0.0001. ns = Non‐significant.

Changes in mitochondrial‐associated proteins, including MFN1, DRP1, OPA1, PGC‐1α and TFAM, were then explored to reveal the underlying differences between Wt and ApoE4. No significant changes were observed in the MFN1, DRP1, OPA1 and TFAM expressions, except PGC‐1α, which was significantly increased in the hippocampus of ApoE4 mice (Figure 5A–F). This finding was further validated by mRNA analysis. Drp1, mfn1 and tfam mRNA levels remained unchanged in ApoE4 mice, except for opa1 mRNA level, which increased in ApoE4 mice (Figure 5G–J). However, melatonin treatment did not change MFN1, DRP1, OPA1 and PGC‐1α expression in ApoE4 mice (Figure 5A–E), except for increased TFAM (Figure 5A,F). Furthermore, melatonin treatment enhanced mfn1 mRNA levels but did not affect drp1, opa1 or mfn2 mRNA levels (Figure 5G–J). Moreover, LPS treatment did not change MFN1, DRP1, OPA1 and PGC‐1α expression (Figure 5K–P) in the ApoE4 mice. However, melatonin treatment significantly reduced OPA1 and increased TFAM levels compared to LPS‐treated ApoE4 mice (Figure 5N,P). Furthermore, we did not find significant changes in the DRP1, MFN1, OPA1 and PGC‐1α expressions in the LPS‐treated ApoE4 mice after UA + AICAR treatment (Figure 5K–P).

### 3.3 LPS‐induced autophagy (mitophagy) impairment in ApoE4 mice

**FIGURE 6:**
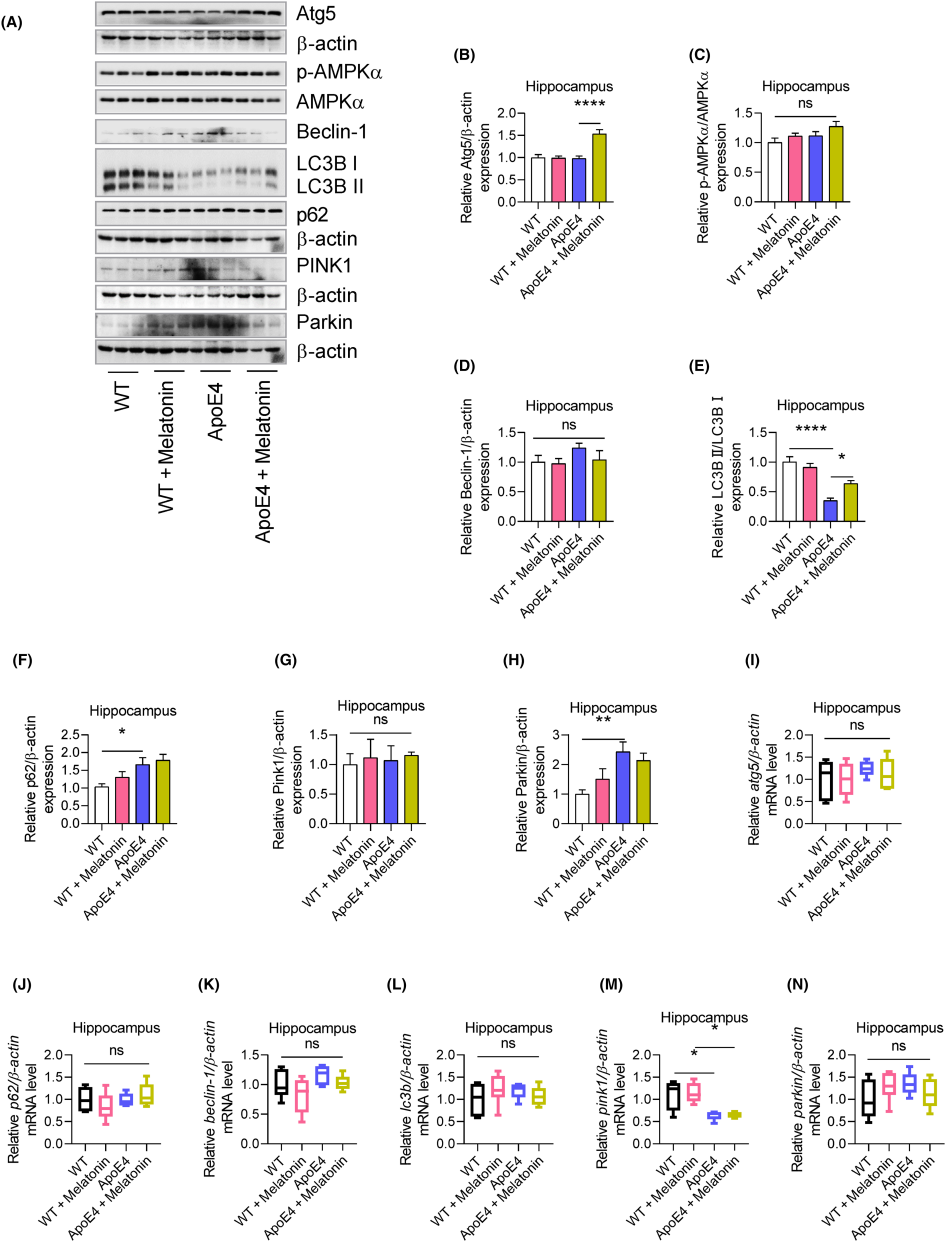
ApoE4 overexpression suppressed autophagy‐related gene expressions. (A–H) Immunoblots images and Bar graphs representing the relative expression of APMK, Atg5, p62, Beclin‐1, LC3B, PINK1 and Parkin in melatonin‐treated WT and ApoE4 mice, *n* = 6. (I–N) Relative mRNA expressions of atg5, p62, beclin‐1, lc3b, pink1 and Parkin in melatonin‐treated WT and ApoE4 mice, *n* = 6. ANOVA, mean ± SEM. *p* < 0.05. *p < 0.05, **p < 0.01, ***p < 0.001, ****p < 0.0001. ns = Non‐significant.

Mitochondrial dysfunction, including impaired mitophagy, contributes to the progression of neuropsychiatric diseases.^48^, ^49^ In this study, we evaluated the potential involvement of mitophagy in the response of ApoE4 mice to lipopolysaccharides (LPS). Autophagy‐associated ATGs and their associated signalling pathways were also measured. ApoE4 mice displayed increased p62, Parkin, but deceased LC3B II expression levels, whereas p‐AMPKα, Atg5, Beclin‐1 and PINK1 remained unchanged (Figure 6A–H). Similarly, no significant changes in atg5, p62, Beclin‐1, lc3b and Parkin mRNAs levels were observed (Figure 6I–N), except for reduced pink1 mRNA levels in ApoE4 mice (Figure 6M). Similarly, PGC‐1α, TFAM and PINK1 were increased, and p‐AMPKα and LC3B II were decreased in HEK 293T cells transfected with ApoE4, whereas Atg5, p62, Beclin‐1 and Parkin were unchanged (Figure S2D,E). Interestingly, melatonin treatment significantly increased Atg5 and LC3B II expression in ApoE4 mice (Figure 6A,C,F), suggesting that ApoE4‐induced impaired mitophagy could be modulated by autophagy owing to melatonin.

**FIGURE 7:**
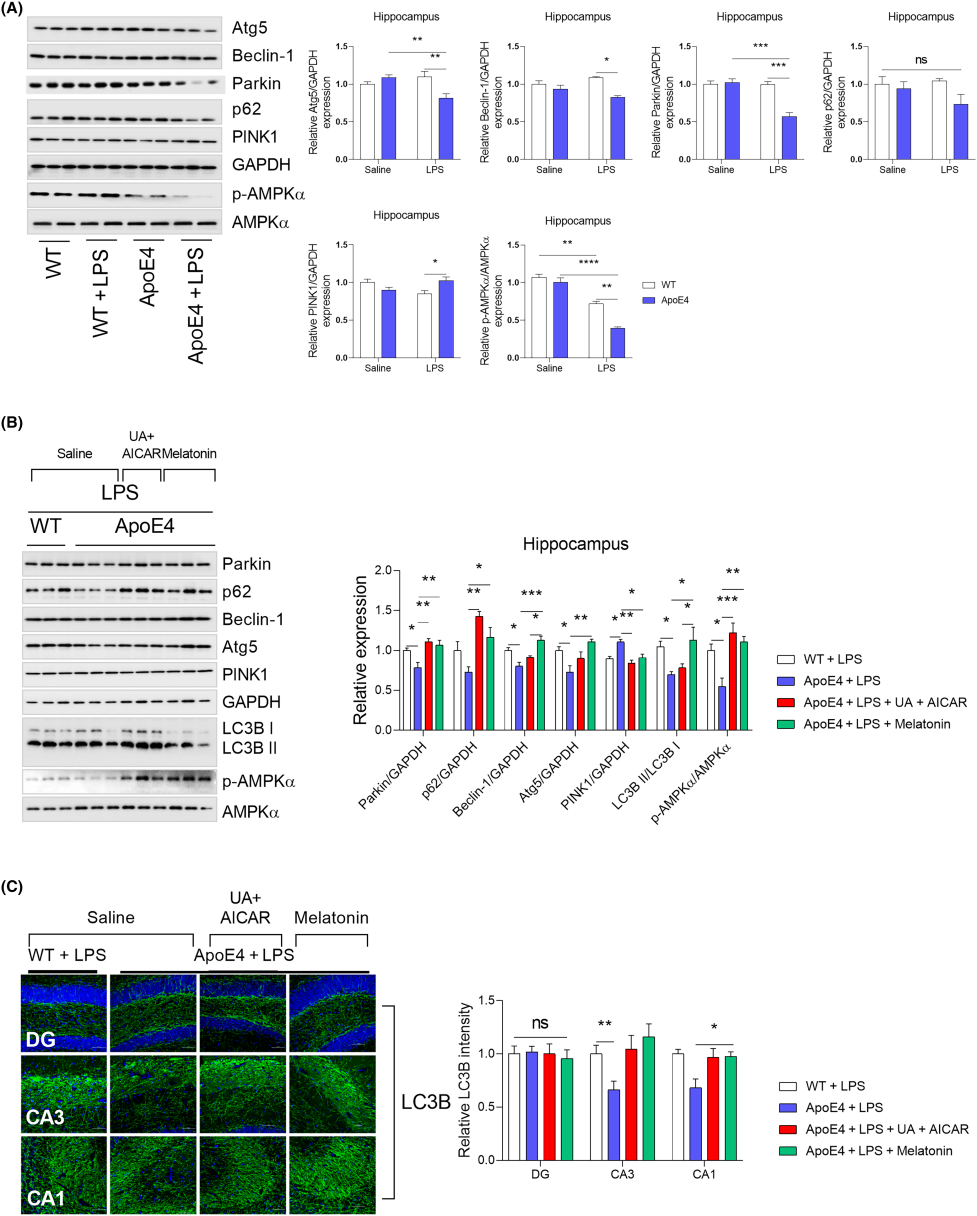
LPS‐suppressed autophagy in ApoE4 mice. (A) Immunoblot images and bar graphs representing the relative expressions of Atg5, p62, Beclin‐1, LC3B, PINK1, Parkin and APMK in melatonin‐treated WT and ApoE4 mice, *n* = 6. (B) Autophagy activators significantly attenuated LPS‐suppressed expression of ATGs, Parkin, p62, Beclin‐1, Atg5, PINK1, LC3B and AMPK, *n* = 6. (C) Representative image of immunofluorescence expressing LC3B in the DG, CA3 and CA1 region of melatonin‐, UA and AICAR‐treated ApoE4 mice in the presence of LPS. ANOVA, post‐hoc Tukey's, mean ± SEM. *p* < 0.05. *p < 0.05, **p < 0.01, ***p < 0.001, ****p < 0.0001. ns = Non‐significant.

Next, we sought to determine whether LPS influences ApoE4‐associated mitophagy impairment. Surprisingly, LPS‐treated ApoE4 mice displayed decreased Atg5, Beclin‐1 and Parkin levels^50^, ^51^ increased PINK1 and p62 levels that remained unchanged (Figure 7A). As an autophagy inducer,^27^, ^52^ melatonin rescued the LPS‐altered expression of ATGs, including Atg5, Beclin‐1 and LC3B II, as well as that of the selective autophagy adapter protein—p62 (Figure 7B). Furthermore, in contrast to PINK1, melatonin treatment significantly increased p‐AMPKα and Parkin expression in ApoE4 mice compared to that in the LPS‐treated ApoE4 mice (Figure 7B,C). These results were further validated using UA + AICAR as an autophagy inducer.^53^, ^54^ Consistent with previous reports, these findings demonstrate that melatonin can improve mitochondrial dysfunction, including mitophagy, as described.^55^, ^56^ Furthermore, ApoE4 was overexpressed in HEK 293T cells (Figure S2A). Similarly, no significant changes in the expression of mitochondrial complex proteins were detected in the ApoE4‐transfected 293T cells (Figure S2B). Except for increased PGC‐1α, TFAM and PINK1, no MFN1, OPA1 and DRP1 changes could be observed in ApoE4 transfected 293T cells (Figure S2C,D).

**FIGURE 8:**
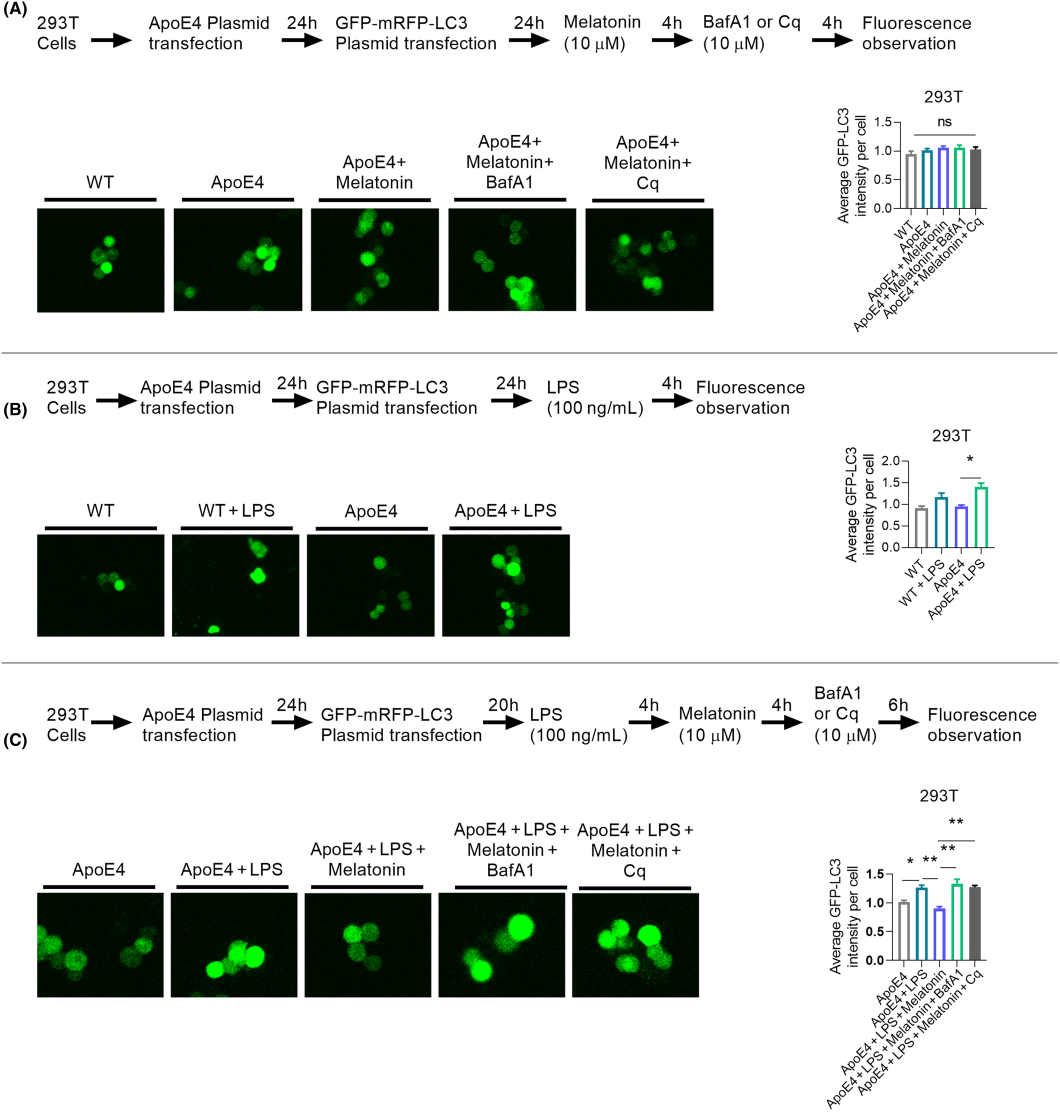
ApoE4 transfection impaired autophagy/lysosomal pathway in HEK 293T cells. (A) Fluorescence observation and GFP‐LC3 intensity in HEK 293T cells after ApoE4 transfection and melatonin, BafA1 and Cq treatment. (B) LPS treatment enhanced GFP‐LC3 intensity in ApoE4 transfected 293T cells. (C) Melatonin treatment reduced GFP‐LC3 intensity, while BafA1 and Cq enhanced GFP‐LC3 intensity in ApoE4‐transfected HEK 293T cells in the presence of LPS. *n* = 3–5, ANOVA, post‐hoc Tukey's, mean ± SEM. *p* < 0.05. *p < 0.05, **p < 0.01, ***p < 0.001, ****p < 0.0001. ns = Non‐significant.

We checked autophagy/lysosomal pathway impairments by further measuring LC‐3 expression in HEK 293T cells using the GFP‐mRFP‐LC3 plasmid to validate mitophagy‐related protein changes. No difference in GFP‐LC3 intensity was observed between the Wt and ApoE4 mice (with or without melatonin/bafilomycin A1: BafA1/Chloroquine: Cq treatment) (Figure 8A). However, LPS treatment significantly enhanced the GFP‐LC3 intensity in ApoE4 mice (Figure 8B), indicating LPS‐induced autophagy/lysosomal pathway impairment. Interestingly, melatonin treatment reduced GFP‐LC3 intensity in HEK 293T cells, suggesting that melatonin could reduce LPS‐induced autophagy/lysosomal pathway impairment. This was further validated by BafA1 and Cq treatments. Both autophagy inhibitors (Baf1A/Cq) significantly enhanced GFP‐LC3 intensity (Figure 8C). Therefore, ApoE4 dysregulation aggravates autophagy (mitophagy) impairment under stress (LPS).

### 3.4 Melatonin receptor antagonism blocked the effects of melatonin

**FIGURE 9:**
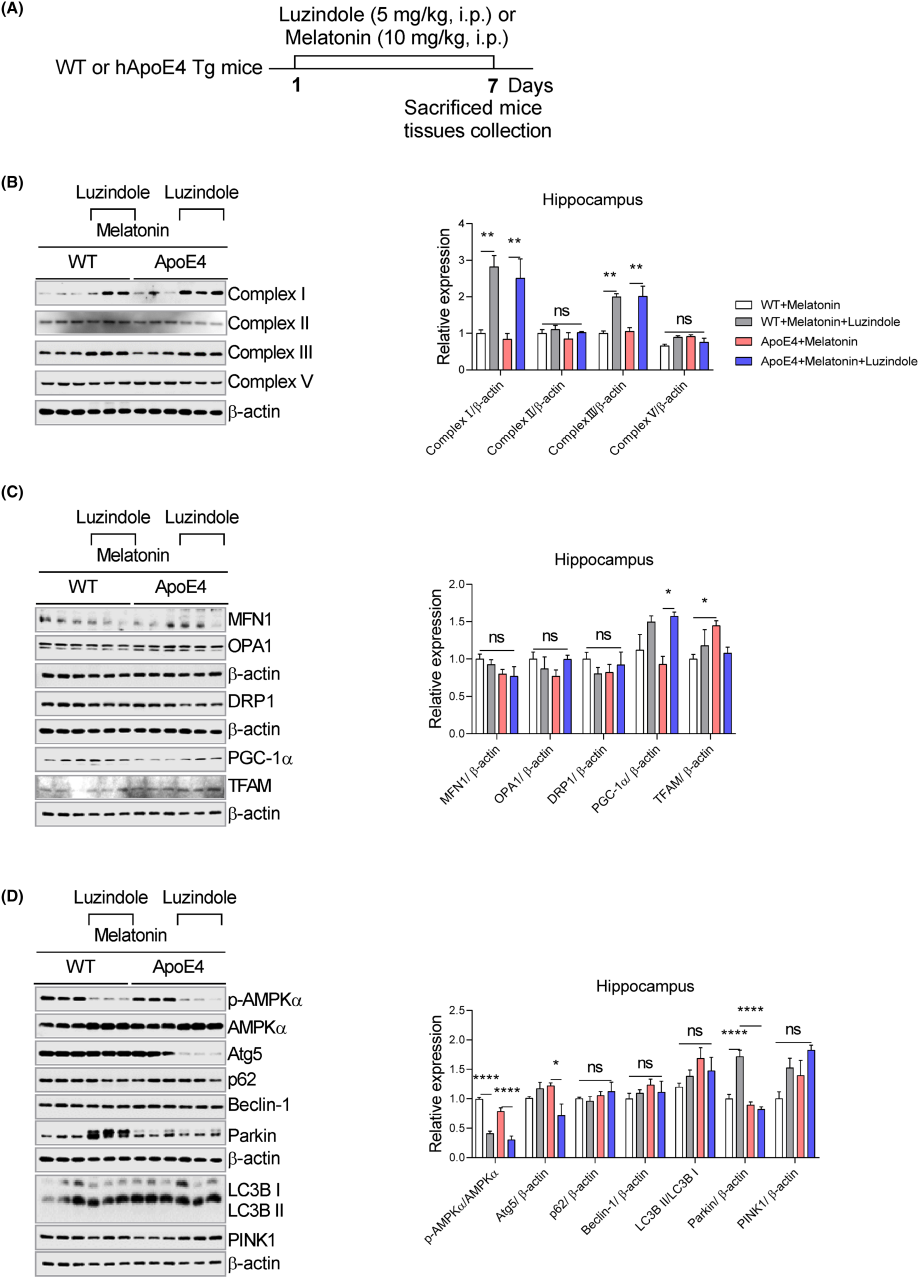
Melatonin receptor antagonism reversed melatonin effects on the mitochondrial‐associated gene expression pattern. (A) Experimental approach, (B) Immunoblots images and bar graphs representing the relative expression of mitochondrial complex, I, II, III and V, *n* = 6. (C) Representative western blot images and their quantitative graphs show the expression of MFN1, OPA1, Drp‐1, PGC1‐α and TFAM, *n* = 6. (D) Immunoblot images and bar graphs representing the relative expression of APMK, Atg5, p62, Beclin‐1, LC3B, Parkin and PINK1 in the luzindole‐ and melatonin‐treated ApoE4 mice hippocampus. *n* = 4–6, ANOVA, pot‐hoc Tukey's, mean ± SEM. *p* < 0.05. *p < 0.05, **p < 0.01, ***p < 0.001, ****p < 0.0001. ns = Non‐significant.

Next, we sought to determine whether the effects of melatonin depended on its receptors. ApoE4 mice were treated with the melatonin receptor antagonist luzindole (Figure 9A). As shown in Figure 9B, luzindole significantly enhanced complexes I and III, whereas complexes II and V remained unchanged. Similarly, luzindole treatment enhanced PGC1‐α expression but not MFN1, OPA1, Drp‐1 and TFAM in the hippocampus of ApoE4 mice (Figure 9C). Concurrently, luzindole treatment reduced Atg5 expression in ApoE4 mice but enhanced Parkin expression in WT mice, whereas a decline of p‐AMPKα expression could be observed in both WT/ApoE4 mice. Moreover, we did not detect significant changes in p62, Beclin‐1, LC3B and PINK1 expression after luzindole treatment (Figure 9D).

Altogether, these results indicated that upon LPS stress, ApoE4 mice exhibited increased neuroinflammation, accelerated free radical generation, impaired mitophagy and led to depression‐like behaviours. Melatonin reversed these detrimental effects of ApoE4 via its receptors.

## 4 DISCUSSION

The present study showed that stress (LPS) can provoke and aggravate neuroinflammation‐associated depression‐like symptoms in ApoE4 transgenic mice. LPS treatment induces depression‐like symptoms and dysregulates cytokine expression in ApoE transgenic mice. Additionally, our in vitro analysis showed increased PGC‐1α and PINK1 but decreased p‐AMPKα and LC3B II in 293T cells transfected with ApoE4. Since PGC‐1α participates in mitochondrial biogenesis,^57^ whereas PINK and Parkin degrade damaged mitochondria via mitophagy,^58^ these results suggest the detrimental effects of the ApoE4 gene on mitochondrial dysfunction. Consequently, melatonin improves mitochondrial dysfunction induced by LPS stress by ameliorating mitophagy impairment. It indicates an interplay between ApoE4 and autophagy impairment under stress conditions. These findings revealed that ApoE4 increased the risk of neuroinflammation‐associated depression via mitophagy impairment, which can be a potential therapeutic target for antidepressants.

Mitochondrial dynamics and dysfunction are considered the hallmarks of neurological disorders^20^; however, the underlying mechanisms are unclear in MDDs. Concurrently, *ApoE4* is a risk factor for neurodegenerative diseases^59^; however, its association with depression has not yet been explored. In our study, ApoE4 elicited mitochondrial dysfunction, further rendering it vulnerable to stimuli, such as LPS, which could be followed by mitophagy deficits, exacerbated neuroinflammation and depression. Furthermore, ApoE4 carriers and transgenic mice demonstrated mitochondrial structural and functional abnormalities, including elevated oxidative and mitochondrial dysfunction, possibly through ApoE4 proteolytic fragments that selectively target mitochondria and impair mitochondrial functions, such as dynamic disturbance, deficits in mitochondrial membrane integrity, defects in electrochemical potential and reduced mitochondrial respiratory activity.^60^, ^61^, ^62^, ^63^, ^64^, ^65^ Previous studies have shown that ApoE4 is associated with impaired astrocyte autophagy and the dysregulation of genes involved in mitochondrial dynamics.^66^ Autophagic signalling is sensitive to the toxic accumulation of proteins and organelles, and its impairment may lead to the toxic accumulation of non‐functional proteins and organelles.^67^, ^68^ Previous studies have demonstrated impaired autophagy pathways in neurological disorders.^69^, ^70^, ^71^ Researchers have also shown a direct link between ApoE4 and coordinated lysosomal expression and regulation (CLEAR) in neurodegenerative diseases, implying a potential role of ApoE4 in impaired autophagy.^9^, ^72^ Furthermore, mitochondrial biogenesis requires mitophagy, which targets damaged or unwanted mitochondria.^73^

Although the mitochondrial complex protein expression did not show alterations in ApoE4, it was dysregulated by LPS administration and improved after melatonin treatment. Parkin is involved in mitophagy.^74^ Our results indicated mitophagy impairment via Parkin dysregulation under stress conditions after ApoE4 overexpression. Our study showed that ApoE4 mice exhibited decreased levels of LC3B II with and without LPS administration, further validating the interplay between autophagy and mitochondria in ApoE4 mice under stress conditions. Additionally, reduced Atg5 expression was observed in ApoE4 mice and an LPS‐induced depression mouse model, delineating the impairment in ApoE4 mice upon stress stimulation.

In our study, melatonin alleviated mitochondrial deficits, eventually reversing neuroinflammation and mitochondrial dysfunction, which may have caused depressive symptoms in ApoE4 mice. Studies have suggested that melatonin can suppress and induce autophagy, including mitophagy,^55^, ^56^, ^75^, ^76^ depending on the experimental conditions. Moreover, melatonin improves mitochondrial biogenesis by regulating mitophagy. However, the exact underlying mechanisms remain poorly understood. The activation of AMPK signalling is considered to be one of the mechanisms involved.^55^ Interestingly, in our results, melatonin treatment recovered suppressed expression of p‐AMPKα as further aggravated by LPS in ApoE4 mice. This confirms that melatonin can improve mitochondrial biogenesis and regulate mitophagy.

Previous studies have shown that neuroinflammation plays a key role in depression‐like behaviours by accelerating synaptic defects.^26^, ^40^ Similarly, patients with MDD have also presented systemic inflammation^77^; however, the mechanism is not precise. Our present and previous studies demonstrated that LPS‐induced neuroinflammation was strongly associated with depression‐like behaviours, which could be attenuated by potent antidepressant‐like melatonin.^40^ Furthermore, astrocytes, microglia and neurons can produce ApoE4,^9^, ^78^ which may act as a proinflammatory factor. LPS administration significantly impaired cytokine levels in ApoE4 transgenic mice. Additionally, LPS‐induced dysregulation of Iba‐1 expression in ApoE4 mice further supports the crucial role of ApoE4 in neuroinflammation. Moreover, our results of enhanced oxidants in ApoE4 mice after LPS administration coincided with previous results showing that ApoE4 microglia produced significantly more NO and proinflammatory factors in mice and humans.^79^, ^80^ These incremental free radicals' origin and detailed mechanisms have yet to be determined; however, melatonin can decrease oxidative stress by scavenging free radicals, followed by reduced neuroinflammation.

## 5 CONCLUSION

Our results revealed that ApoE4 might be associated with depression severity via inflammatory signalling. It can act as a proinflammatory factor and accelerate neuroinflammation‐associated depression‐like behaviours. These changes may be associated with impaired autophagy. Thus, these findings offer a promising mechanistic and therapeutic avenue for the development of antidepressants through the regulation of autophagy.

## AUTHOR CONTRIBUTIONS

**Weifen Li:** Formal analysis (lead); investigation (lead); methodology (lead). **Tahir Ali:** Conceptualization (equal); data curation (equal); formal analysis (lead); writing – original draft (lead); writing – review and editing (lead). **Kaiwu He:** Methodology (equal). **Chengyou Zheng:** Methodology (equal). **Ningning Li:** Funding acquisition (equal); methodology (equal); resources (equal); visualization (equal). **Zhi‐Jian Yu:** Funding acquisition (equal); project administration (equal); resources (equal); visualization (equal). **Shupeng Li:** Conceptualization (lead); funding acquisition (lead); project administration (lead); supervision (lead); validation (lead).

## FUNDING INFORMATION

This work was supported by Grants Science and Technology Innovation Committee of Shenzhen No: JCYJ20170810163329510; Guangdong Basic and Applied Basic Research Foundation (2022A1515010979); National Natural Science Foundation of China (81902033; 82002137); Sanming Project of Medicine in Shenzhen (No. SZSM201911003); Science, Technology and Innovation Commission of Shenzhen Municipality of Basic Research Funds (JCYJ20190809110209389; JCYJ20190809102219774); Shenzhen Key Medical Discipline Construction Fund (No.SZXK06162); Shenzhen Nanshan District Major Scientific Research Program of the People's Republic of China (NSZD2023019); Shenzhen‐Hong Kong Institute of Brain Science‐Shenzhen Fundamental Research Institutions No: 2019SHIBS0004.

## CONFLICT OF INTEREST STATEMENT

The authors have no conflicts of interest to declare.

## Supporting information


Data S1.

Figures S1–S2.

## Data Availability

All data generated or analysed in this study are included in the published article [and its supplementary information files].
