# Supplementary material for: ApoE4 dysregulation incites depressive symptoms and mitochondrial impairments in mice
Source: J Cell Mol Med. 2024 Mar 20;28(7):e18160. doi: 10.1111/jcmm.18160 (PMC10951871; doi:10.1111/jcmm.18160)
Supplement: Supplementary file 1 — Data S1. [file JCMM-28-e18160-s001.docx]

Supplementary data

**Open field test (OFT)**

To eliminate the animal sickness factors and avoid the biases due to sickness and blunted behaviors induced by LPS, we performed OFT analysis according to previously developed protocols^24^. Briefly, mice were adapted to the experimental room for 1h and placed in the 45× 45 ×30 cm chamber. A total of 15min video was recorded to observe the mice's locomotor activity. The total distance covered by mice was measured, analyzed, and expressed in meters.

**Sucrose preference test**

A sucrose preference test was performed using a two-bottle free-choice paradigm. Mice were habituated with a 1% sucrose solution for three days and then randomly grouped. Mice were deprived of water and food for 24 hours on the three days of drug administration to assess the individual sucrose intake. Each mouse had free access to two bottles containing sucrose and water the next day. The water and sucrose-containing bottle positions were changed after 12 hours. Finally, the volume of consumed water and sucrose solution were recorded and calculated by the following formula:

$$SPT=\frac{Sucrose consumption}{water and Sucrose consumtion}\times100\%$$

**Forced Swimming Test (FST)**

The forced swimming test (FST) was performed according to our previously developed protocols^25^. First, the experimental animals were trained for swimming and performed pre-experiment FST to select healthy and normal mice. During FST, mice were placed in a Plexiglas cylinder (height: 70 cm, diameter: 30 cm) filled with water over the 30cm level at a temperature of 23 ± 1 °C. In a total of 6 minutes of video, the last 5 minutes were blindly analyzed. Mice considered immobile when they remained floating motionless in the water and just making a move to keep their nose above the water surface. The animals’ horizontal movement throughout the cylinder was defined as swimming, while the vertical direction against the cylinder’s wall was climbing. EthoVision XT was used to record the video and analyze the results.

**Tail suspension test (TST)**

The tail suspension test was performed as described previously^24,26^. Briefly, the mice were individually suspended about 40 cm above the floor by their tail with the tape in the rectangular compartment (55height × 20 widths × 11.5 cm depth). The immobility time was scored for the first 4min of the total 5-min video. EthoVision XT software was used to record and analyze the data.

**Nitric oxides and H_2_O_2_ Measurement, and TBARs assays**The level of NO and H_2_O_2_ was analyzed by a commercially available kit (Beyotime Institute of Biotechnology, China, CAT# S0021M, and CAT# S0038, respectively) ^28,29^.

Briefly, for the NO detection: 20μL of the sample (serum/homogenates) were added in the mixture of Reagents (Gryess Reagent) R1+R2 (100 μL+ 100 μL), and absorbance recorded at 540nm. Similarly, for the H_2_O_2_ detection, 20μL sample (serum/homogenates) was added in the 100 μL of the detection reagent, and after 30min room temperature incubation, the absorbance was recorded at 560nm.

**TBARs assay**

TBARS levels are used to determine the damage to lipids caused by reactive oxygen species in various experimental groups^30,31^. Briefly, 0.1 ml of sample, 0.1 ml FeSO4, 0.1 ml Tris-HCl, 0.6 ml distilled water, and 0.1 ml Ascorbic Acid were incubated at 37 °C in a test tube 15 minutes, and then 1 ml TCA and 2 ml TBA were added. These plugged test-tubes incubated for 15 minutes at 100 °C, followed by centrifugation at 3000 rpm for 10 minutes. The supernatant O.D. was determined at 532 nm and the following formula was applied to estimate TBARS as nM/mg protein: TBARS (nM/mg protein) = O.D × Total volume× Sample volume × 1.56 × 105 × mg protein/ml (1.56 × 105 = Molar Extinction Coefficient)

**Proteomic Analysis**

The proteomic analysis was performed as we performed previously ^27^ and used DAVID version 6.7 (https://david.ncifcrf.gov/) to classify the functional categories. Briefly, we followed the below steps, 1); Protein Extraction and Digestion：Hippocampus tissue samples from WT and hApoE4 mice were isolated and frozen in liquid nitrogen and stored at −80 °C until use. 2); Tandem Mass Tag (TMT) Labeling. 3); Peptide Fractionation with High pH Reversed-Phase Fractionation. 4); NanoLC−MS/MS, and Database Searching. 5; Bioinformatic Analysis.

**Western blots**

Brain hippocampal tissues were homogenized in RIPA lysis buffer with protease inhibitor cocktail (MedChem Express Monmouth Junction, NJ, USA), followed by shaking at 4 °C for 30 min to promote lysis. The sample was then spun down for 10 min at 12,000rpm. Homogenized protein samples were quantified using a Bradford protein assay, and 15 ug protein loaded per well. Samples were separated by size in SDS-page running buffer and transferred onto a PVDF membrane to a solution of Tris-Glycine transfer buffer, 20% methanol, and 0.1% SDS. Membranes were then washed in TBS-T (1x TBS, 0.1% Tween-20) and transferred to blocking buffer (2% BSA, 5% milk, 1x TBS-T) for one h at RT. Primary antibodies diluted in carrier solution (Can get signal, Solution 1, NKB-201, Japan), followed by incubation at 4 °C O/N on a shaker. Blots were then washed three times for 5 min each in TBS-T at R/T. Secondary HRP-conjugated antibodies (Santa Cruz) were all diluted 1:10,000 in carrier solution (5% non-fat milk diluted in TBST), and blots were incubated for 1hr at R/T on a shaker. Membranes were washed three times in TBST and then incubated for 1 min in ECL (Thermo Scientific). Blots developed using Chemidoc mp Bio-red. The densitometry analysis of the bands performed through image lab software. The band size and density measurements from each sample were collected using ImageJ. Values were normalized to loading controls (β-actin and GAPDH) and reported as a proportion of loading control expression.

**ELISA**

Frozen hippocampal and cortical tissue was lysed with RIPA buffer and homogenized on ice. Supernatants were collected after centrifugation and stored at freezing temperature for further analysis. ELISA kits (ABclonal) were used according to the manufacturer’s protocols to quantify cytokine expression. Briefly, after washing the wells of the 96-well plate, 100 µL standard/sample was added and incubated for 2 hours at 37 ̊°C. Next, the plates were cleaned, and each well was added with a biotin-conjugated antibody (1:30), followed by incubation for 1 hour at 37 ̊°C and Streptavidin-HRP for 30 minutes at 37 ̊°C. Finally, the reaction was stopped, and the optical density was measured accordingly.

**Mitochondrial copy number**

For relative mitochondrial copy number (mtCN) estimation, qPCR based DNeasy Blood and Tissue Kit (Qiagen Ltd, UK) was used. Primer sequences for the mitochondrial segment were as follows: (F) GCCAGCCTGACCCATAGCCATAAT and (R) GCCGGCTGCGTATTCTACGTTA. Primer sequences for the single-copy nuclear control were as follows: (F) TTGAGACTGTGATTGGCAATGCCT and (R) CCAGAAATGCTGGGCGCTCACT. The mtCN was calculated relative to nuclear DNA using the following equations: 1. ΔC_T_ = mitochondrial C_T_ – nuclear C_T;_ 2. Relative mitochondrial DNA content = 2 X 2^_ΔCT^

**ATP assay**

ATP assays performed according to the instructions provided with ATP analysis ki (Cat # S0026, Beyotime technology, China). Briefly, the hippocampus of mice was split with ATP lysate, boiled at 100 ° C for two minutes, centrifugated at 4 ° C at 12000rpm for 5min, and collected the supernatant. Next, 50uL of the sample was added into 100uL of the reagents working solution and finally measured by the luminometer.

**Immunofluorescence**

Immunofluorescence staining was performed according to previously reported protocols^32^. Firstly, 20 μm brain sections were washed with PBS for 15 minutes (5 min ×3), followed by blocking with buffer (10% Goat serum in 0.3% Triton X-100 in PBS) for 1 hour at room temperature. After blocking, the tissue was treated with primary antibodies (Iba1, GFAP, and LC3 B) overnight at 4̊ °C. The next day, secondary antibodies (Alexa Flour secondary antibodies, ThermoFisher) were applied at room temperature for 1 hour. The sections were washed with PBS for 5 minutes three times. After washing, sections were transferred to slides, and glass coverslips were mounted using the mounting medium, followed by images developing under inverted fluorescence microscope IX73 Olympus.
