# Supplementary material for: ApoE4 dysregulation incites depressive symptoms and mitochondrial impairments in mice
Source: J Cell Mol Med. 2024 Mar 20;28(7):e18160. doi: 10.1111/jcmm.18160 (PMC10951871; doi:10.1111/jcmm.18160)
Supplement: Supplementary file 2 — Figures S1–S2. [file JCMM-28-e18160-s002.docx]

**Figure S1: LPS accelerated free radicals generation in ApoE4 mice.**

**(A-F):** Bar graphs representing ROS, H_2_O_2_, and Nitrite level in serum and hippocampus, n=7, mean ±SEM, ANOVA, Tukey's test. **(G-I):** Bar graphs showing serum H_2_O_2_, ROS, and NO, levels in UA, AICAR, melatonin, and LPS treated aApoE4 mice. n=8, mean ±SEM, ANOVA, Tukey's test.

**Figure S2: ApoE4 triggered mitochondrion and autophagy dysfunction.**

**(A):** Drug treatment schedule, **(B-E):** Immunoblots images and collum graphs representing the relative expression of Complex I, II, III, and V, OPA1, DRP1, MFN1, PGC-1α, TFAM, Parkin, PINK1, p-AMPKα, AMPKα, ApoE4, Atg5, Beclin-1, p62, LC3B I and II, in the 293T Cells.
